# Combination of midbrain-to-pontine ratio and cardiac MIBG scintigraphy to differentiate Parkinson's disease from multiple system atrophy and progressive supranuclear palsy

**DOI:** 10.1016/j.prdoa.2019.12.002

**Published:** 2019-12-11

**Authors:** Hirotaka Sakuramoto, Hiroaki Fujita, Keisuke Suzuki, Takeo Matsubara, Yuji Watanabe, Mai Hamaguchi, Koichi Hirata

**Keywords:** Parkinson's disease, Multiple system atrophy, Progressive supranuclear palsy, MRI methodology, Midbrain-to-pontine ratio, Cardiac ^123^I-metaiodobenzylguanidine (MIBG) scintigraphy

## Abstract

**Background:**

An early clinical differentiation between Parkinson's disease (PD) and multiple system atrophy (MSA) or progressive supranuclear palsy (PSP) remains a challenge. The purpose of this study was to evaluate the usefulness of the combination use of midbrain-to-pontine ratio (M/P ratio) from magnetic resonance imaging (MRI) with cardiac ^123^I-metaiodobenzylguanidine (MIBG) uptake for differentiating PD from MSA and PSP.

**Methods:**

Ninety-six parkinsonian patients (70 PD, aged 68.5 ± 9.5 years; 16 MSA, aged 67.9 ± 7.5 years; 10 PSP, aged 70.4 ± 9.4 years) who underwent MRI and cardiac MIBG scintigraphy were included in this study. Receiver operating characteristic (ROC) curve analysis was used to assess the sensitivity and specificity for distinguishing PD from MSA and PSP patients. The diagnostic accuracy of these tests was also assessed among patients at the early disease stage (defined as patients with a disease duration of 3 years or less).

**Results:**

The individual diagnostic sensitivity of the M/P ratio and cardiac MIBG scintigraphy was 87.1% and 67.1% in PD vs. MSA and 78.6% and 67.1% in PD vs. PSP, respectively. The diagnostic specificity of the M/P ratio and cardiac MIBG scintigraphy was 56.3% and 100% in PD vs. MSA and 70.0% and 90% in PD vs. PSP, respectively. With the optimal cutoff values, at least one positive result (either the M/P ratio or cardiac MIBG revealed abnormalities) improved sensitivity (95.7%) without decrease of specificity (56.3%) in PD vs. MSA, as well as in PD vs. PSP (100% sensitivity, 70.0% specificity). In contrast, both positive results of two tests had good specificity but low sensitivity in PD vs. MSA (60.0% sensitivity and 100% specificity) and in PD vs. PSP (47.1% sensitivity and 90% specificity). Similar trends were observed in early-stage patients.

**Conclusion:**

Although M/P ratio alone was potentially useful for distinguishing PD from MSA or PSP, the combined use with cardiac MIBG scintigraphy can further improve the diagnostic accuracy of PD from MSA or PSP.

## 1 Introduction

Parkinson's disease (PD) is the second most common neurodegenerative disorder after Alzheimer's disease and is now the fastest growing neurological disorder [1]. Considering the emerging evidence of a PD pandemic [1] and the negative impact on quality of life, especially when patients are left untreated [2], early diagnosis and treatment are imperative. However, the differential diagnosis of PD based on neurological findings alone remains difficult, particularly in the early disease stage, during which atypical parkinsonian syndromes, including progressive supranuclear palsy (PSP) and multiple system atrophy (MSA), often mimic the clinical manifestations of PD. Thus, the accurate diagnosis of PD currently represents a challenge for neurologists, and several clinical markers have been described to enhance diagnostic accuracy [[3], [4], [5]], but some of them can be influenced by common comorbidity of PD, such as dementia. There are two following imaging test which can be used even individuals with cognitive impairment.

Cardiac ^123^I-metaiodobenzylguanidine (MIBG) scintigraphy, which assesses cardiac sympathetic nerve function, is also reportedly beneficial in differentiation between PD and MSA or PSP. MIBG uptake is significantly reduced in patients with PD, while its uptake is preserved in patients with MSA and PSP [[6], [7], [8]]. Cardiac sympathetic denervation on cardiac MIBG scintigraphy has been included as supportive criteria in the Movement Disorder Society (MDS) diagnostic criteria for PD [9]. We have previously reported that at least 2 abnormal results from substantia nigra hyperechogenicity, cardiac MIBG scintigraphy, and olfactory testing contributed to a better differential diagnosis of PD from MSA and PSP [7].

Selective midbrain atrophy, well known as the hummingbird sign or morning glory sign, has recently received much attention as a supportive diagnostic marker of PSP [10]. On the other hand, pontine atrophy is pronounced in MSA. Several previous studies have shown that the midbrain-to-pontine ratio (M/P ratio) calculated using magnetic resonance imaging (MRI) might be a candidate supportive marker for differentiating PD from PSP and MSA [[11], [12], [13], [14]]. However, there has been no study evaluating the usefulness of the M/P ratio for PD diagnosis in combination with other imaging modalities. In this study, we aimed to evaluate the utility of the M/P ratio in combination with cardiac MIBG for differentiating PD from MSA and PSP patients.

## 2 Methods

This cross-sectional study was performed in accordance with the Declaration of Helsinki and approved by the institutional review board of Dokkyo Medical University. All participants provided written informed consent.

### 2.1 Subjects

From April 2016 to March 2019, patients with PD, MSA and PSP in whom MRI and cardiac MIBG scintigraphy were performed were enrolled in this study. A total of 96 parkinsonian patients (PD, 70 (clinically probable PD, 49; clinically possible PD, 21); MSA-C, 8; MSA-P, 8; PSP-Richardson Syndrome (PSP-RS), 8; and PSP-progressive gait freezing (PSP-PAGF), 2) were included in this study. Diagnoses of PD, MSA, and PSP were made based on the MDS-PD criteria [9], second consensus statement on the diagnosis of MSA [15], and MDS PSP criteria [16], respectively. A total of 84.4% of the patients underwent a dopamine transporter (DAT) scan to assess presynaptic dopaminergic dysfunction. All the patients were followed for at least 3 years after disease onset to confirm the initial diagnosis. For patients in whom disease duration was <3 years at the time of study were additionally follow-up at outpatient clinic for at least 3 years after disease onset and no patient changed initial diagnosis. We analyzed subgroups of early-stage patients, who were defined as patients with a disease duration of 3 years or less. In the subanalysis of the M/P ratio in MSA, the difference between the MSA-P and MSA-C patients was compared.

### 2.2 Clinical assessments

Disease severity was rated using the Hoehn and Yahr (HY) stage [17]. Cognitive function was assessed by the Mini-Mental State Examination (MMSE) Japanese version [18,19]. Levodopa equivalent doses were calculated according to previously described methods [20].

### 2.3 Midbrain-to-pontine ratio

Midsagittal sections from T1-weighted MRI images were used. Two straight lines were drawn. The first line was drawn to pass through the superior pontine notch and inferior edge of the quadrigeminal plate. The second line was drawn parallel to the first line to pass through the inferior pontine notch. The area of the midbrain was traced around the edges of the first line and the delta-shaped midbrain tegmentum above it. The area of the pontine was the area inside the line traced along the anterior and posterior margins of the pontine and along the first line and the second line, as previously described [12].

### 2.4 Cardiac ^123^I-metaiodobenzylguanidine scintigraphy

Chest SPECT and planar images were obtained using a gamma camera 15 min (early phase) and 4 h (delayed phase) after intravenous injection of 111 MBq ^123^I-MIBG (Fujifilm RI Pharma Co., Tokyo, Japan) into each patient in the supine position. The heart-to-mediastinum (H/M) ratio was calculated by dividing the count density of the left ventricular region of interest (ROI) by that of the mediastinal ROI, as previously described [21]. In this study, the delayed phase H/M values were used. During the study period, machines of MRI and cardiac MIBG scintigraphy were not changed.

### 2.5 Statistical analyses

The Kruskal-Wallis test was used as appropriate to compare the continuous variables. *P*-values were corrected according to Bonferroni. To compare the categorical variables among groups, the chi-square test was applied. Based on receiver operating characteristic (ROC) curves, the sensitivity and specificity were calculated to determine the optimal cutoff values of the M/P ratio and H/M ratio for differentiating PD from MSA and PSP. Spearman rank correlation coefficients were used to assess correlations. The M/P ratio of 20 patients was randomly determined by H.S. at a mean interval of 7 days, and test-retest reliability (intrarater reliability) was assessed by intraclass correlation coefficients (ICC, one-way random effects model). The M/P ratios were determined by 2 neurologists, H.S. and T.M., blind to clinical diagnosis, and interrater reliability was assessed by ICC (two-way random effects model). A *P*-value < 0.05 was considered statistically significant. Analyses were performed by SPSS Statistics, Version 25 (IBM SPSS, Tokyo, Japan). GraphPad Prism for Windows (Version 5.01; GraphPad Software, San Diego, USA) was used for the figures and ROC curve analyses.

## 3 Results

Nine of 16 MSA patients (56.3%) showed decrease in striatal DAT uptake. Among 8 MSA-C patients, 5 (62.5%) showed abnormality. The clinical characteristics of the patients with PD, MSA and PSP are shown in Table 1. There were no significant differences in age or sex among the three groups. The disease severity rated by the HY stages did not show significant differences among the groups. The percentages of de novo patients in the PD, MSA and PSP groups were 41.4%, 75.0%, and 40.0%, respectively. The delayed H/M ratio of cardiac ^123^I-MIBG uptake (PD: 1.99 ± 0.89, MSA: 3.23 ± 0.54, PSP: 2.92 ± 0.76; *p* < 0.001) was significantly lower in the patients with PD than in those with MSA and PSP. The intrarater reliability and interrater reliability (ICC) for the M/P ratio were high (0.90 and 0.93, respectively). The M/P ratio showed significant differences among the three groups (PD: 0.238 ± 0.032, MSA: 0.292 ± 0.078, PSP: 0.192 ± 0.043; *p* < 0.001). In the subanalysis of the M/P ratio that included only MSA patients, the MSA-P patients showed a significantly lower M/P ratio than MSA-C patients (0.25 ± 0.53 vs. 0.34 ± 0.77; *p* = 0.016).

**Table 1:** Background characteristics of the patients.

	PD	MSA	PSP	p-Value
Age (y)	68.5 ± 9.5	67.9 ± 7.5	70.4 ± 9.4	0.791
Onset age (y)	64.6 ± 10.5	65.0 ± 8.3	66.9 ± 9.0	0.800
Sex (M/F)	29/41	9/7	4/6	0.252
Disease duration (y)	3.9 ± 4.0	2.9 ± 3.0	3.6 ± 3.2	0.661
HY stage				0.010
Stage 1 (%)	5 (7.1)	1 (6.2)	0 (0)	
Stage 2 (%)	30 (42.9)	3 (18.8)	1 (10.0)	
Stage 3 (%)	27 (38.6)	8 (50.0)	5 (50.0)	
Stage 4 (%)	4 (5.7)	2 (12.5)	2 (20.0)	
Stage 5 (%)	4 (5.7)	2 (12.5)	2 (20.0)	
De novo, n (%)	29 (41.4)	12 (75.0)	4 (40.0)	0.007
MMSE	26.5 ± 3.3	25.1 ± 4.0	26.2 ± 3.6	0.390
Cardiac MIBG scintigraphy (H/M)	1.99 ± 0.89	3.23 ± 0.54⁎	2.92 ± 0.76⁎	<0.001
LED (mg/day)	257.0 ± 336.1	72.5 ± 158.6⁎	385.0 ± 448.5	0.047
Midbrain area (mm2)	132.2 ± 20.3	124.7 ± 14.0	98.6 ± 23.4⁎, ¶	<0.001
Pons area (mm2)	558.8 ± 61.2	452.0 ± 102.7⁎	512.4 ± 32.7	<0.001
M/P ratio	0.238 ± 0.032	0.292 ± 0.078⁎	0.192 ± 0.043⁎, ¶	<0.001

PD = Parkinson's disease, MSA = multiple system atrophy, PSP = progressive supranuclear palsy, HY=Hoehn and Yahr, MMSE = Mini-Mental State Examination, MIBG = metaiodobenzylguanidine, LED = levodopa equivalent dose.

⁎ p < 0.05, compared with PD.

¶ p < 0.05, compared with PSP.

**Fig. 1:**
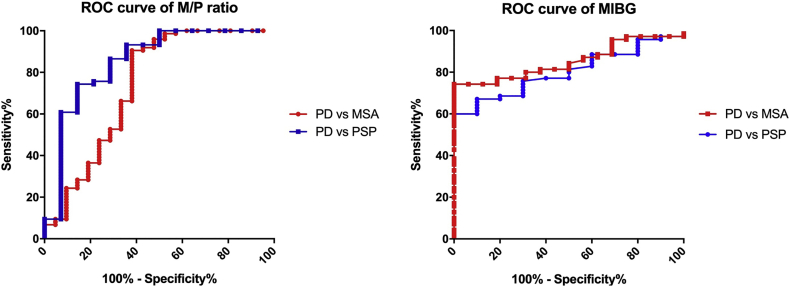
Receiver operating characteristic curves for the M/P ratio and cardiac MIBG scintigraphy for discriminating PD vs. MSA and PD vs. PSP.

Fig. 1 shows ROC curves for the M/P ratio and cardiac MIBG scintigraphy in PD vs. MSA and PD vs. PSP, respectively. The area under the ROC curves (AUC) for the M/P ratio and cardiac MIBG scintigraphy in the PD vs. MSA comparison were 0.74 (95% CI, 0.59–0.89; *p* < 0.001) and 0.85 (95% CI, 0.77–0.93; p < 0.001), respectively. The AUCs for the M/P ratio and cardiac MIBG scintigraphy in the PD vs. PSP comparison were 0.85 (95% CI, 0.72–0.98; p < 0.001) and 0.80 (95% CI, 0.70–0.91; *p* = 0.002), respectively. According to the ROC curve, we determined the optimal cutoff points to differentiate PD vs. MSA and PD vs. PSP as follows. For PD vs. MSA, the cutoff points were an M/P ratio < 0.28 (87.1% sensitivity and 56.3% specificity) and cardiac MIBG scintigraphy (delayed H/M ratio) <2.00 (67.1% sensitivity and 100% specificity). For PD vs. PSP, the cutoff points were an M/P ratio > 0.21 (78.6% sensitivity and 70.0% specificity) and cardiac MIBG scintigraphy <2.00 (67.1% sensitivity and 90% specificity).

Table 2 summarizes the sensitivity and specificity of the M/P ratio and cardiac MIBG scintigraphy in PD vs. MSA and PD vs. PSP with different combinations of test batteries. Compared with the results from each individual test (option 1), at least one positive result from two tests (option 3) improved the sensitivity for differentiating PD vs. MSA (95.7%) and PD vs. PSP (100%), without decease of specificity (56.3% and 70%). In contrast, both positive results from two tests (option 2) improved the specificity for differentiating PD vs. MSA (100%) and PD vs. PSP (90%) but low sensitivity (60% and 47.1%, respectively).

**Table 2:** Positive ratio, sensitivity and specificity of M/P ratio and cardiac MIBG scintigraphy and their combination in diagnosing PD versus MSA and PSP groups.

(A) PD vs. MSA						
Option		PD	MSA	p-Value	PD diagnosis	
					Sensitivity (%)	Specificity (%)
1	M/P ratio (<0.28)	61 (87.1%)	7 (43.8%)	<0.001	87.1	56.3
	Cardiac MIBG (<2.0)	47 (67.1%)	0 (0%)	<0.001	67.1	100
2	Positive results in both	42 (60.0%)	0 (0%)	<0.001	60.0	100
3	At least one positive result from the 2 tests	67 (95.7%)	7 (43.8%)	<0.001	95.7	56.3


(B) PD vs. PSP						
Option		PD	PSP	p-Value	PD diagnosis	
					Sensitivity (%)	Specificity (%)
1	M/P ratio (>0.21)	55 (78.6%)	3 (30.0%)	0.001	78.6	70.0
	Cardiac MIBG (<2.0)	47 (67.1%)	1 (10%)	0.001	67.1	90.0
2	Positive results in both	33 (47.1%)	1 (10%)	0.026	47.1	90.0
3	At least one positive result from the 2 tests	70 (100%)	3 (30%)	<0.001	100	70.0

The subanalysis of early-stage patients (*n* = 63) showed similar trends as those observed in the total cohort (Table 3). Although the sensitivities of the M/P ratio and cardiac MIBG scintigraphy alone (option 1) were relatively low in PD vs. MSA (89.1% and 67.4%) and in PD vs. PSP (76.1% and 67.4%), the combined use of both tests (option 3) improved the sensitivity with equal specificity (95.7% and 63.6% in PD vs. MSA, 100% and 83.3% in PD vs. PSP, respectively).

**Table 3:** Positive ratio, sensitivity and specificity of M/P ratio and cardiac MIBG scintigraphy and their combination in diagnosing early PD versus early MSA and PSP groups.

(A) Early PD vs. early MSA						
Option		PD	MSA	p-Value	PD diagnosis	
					Sensitivity (%)	Specificity (%)
1	M/P ratio (<0.28)	41 (89.1%)	4 (36.4%)	<0.001	89.1	63.6
	MIBG (<2.0)	31 (67.4%)	0 (0%)	<0.001	67.4	100
2	Positive results in both	28 (60.9%)	0 (0%)	<0.001	60.9	100
3	At least one positive result from the 2 tests	44 (95.7%)	4 (36.4%)	<0.001	95.7	63.6


(B) Early PD vs. early PSP						
Option		PD	PSP	p-Value	PD diagnosis	
					Sensitivity (%)	Sensitivity (%)
1	M/P ratio (>0.21)	35 (76.1%)	1 (16.7%)	0.003	76.1	83.3
	MIBG (<2.0)	31 (67.4%)	0 (0%)	0.002	67.4	100
2	Positive results in both	20 (43.5%)	0 (0%)	0.040	43.5	100
3	At least one positive result from the 2 tests	46 (100%)	1 (16.7%)	<0.001	100	83.3

The M/P ratio was weakly negatively correlated with age (*r* = −0.29, *p* < 0.05) in the PD group and was positively correlated with MMSE scores in the PD and MSA groups (*r* = 0.32, *p* < 0.01 and *r* = 0.60, p < 0.01, respectively). Cardiac MIBG uptake was negatively correlated with age and HY stage (*r* = −0.25, p < 0.05 and r = −0.29, *P* < 0.05) in the PD group. There was no correlation between the M/P ratio and cardiac MIBG reuptake in any group.

## 4 Discussion

We evaluated the usefulness of the combination use of M/P ratio with cardiac MIBG scintigraphy for differentiating PD from MSA and PSP. The present study confirmed that the M/P ratio alone was the potentially useful tool for differentiating PD from MSA and PSP, but relatively low sensitivity was the problem to use in practice. The present study demonstrated that the combination use of M/P ratio with cardiac MIBG scintigraphy could substantially improve its diagnostic power.

Although the M/P ratio is not included in the MDS clinical diagnostic criteria for PD [9], some previous reports have suggested the utility of the M/P ratio for diagnosing parkinsonian syndromes. Oba et al. [12] reported that the M/P ratio of the PSP group (0.124 ± 0.15) was significantly lower than that of the PD group (0.208 ± 0.031), MSA-P group (0.266 ± 0.067) and normal control group (0.236 ± 0.034). Constantinides et al. [11] reported the utility of M/P ratio and suggested the optimal cutoff value. In the current study, the sensitivity and specificity of the M/P ratio for discriminating PD vs. MSA (option 1) were 87.1% and 56.3% (with a cutoff value of 0.28), and the sensitivity and specificity for discriminating PD vs. PSP were 78.6% and 70.0% (with a cutoff value of 0.21). Although M/P ratio individually showed usefulness to differentiate PD from MSA and PSP, the combined use with cardiac MIBG scintigraphy improved sensitivity. At least one positive result (option 3) for the M/P ratio or cardiac MIBG scintigraphy showed improved sensitivity (95.7% in PD vs. MSA, 100% in PD vs. PSP) without decrease of specificity (56.3% and 70%, respectively). In early-stage patients, although the sensitivity of the M/P ratio alone (option 1) was relatively low, especially in PD vs. PSP (76.1%), the combined use of both tests (option 3) improved the sensitivity (95.7% in PD vs. MSA, 100% in PD vs. PSP) without worsening of specificity (63.6% and 83.3%, respectively). In the subanalysis of the M/P ratio, patients with MSA-P had significantly lower values than patients with MSA-C, which was consistent with a previous report [11] and is in accordance with clinical features.

There were some previous reports that the M/P ratio might be influenced by clinical features. Morelli et al. [22] reported that age was negatively correlated with the M/P ratio in healthy controls and patients with PD. Oba et al. [12] reported that the M/P ratio correlated with disease duration in patients with PSP but did not correlate with patient sex or age at the time of the MRI study. Our study showed that the M/P ratio was negatively correlated with age in the PD group, but it was not correlated with sex, disease duration or disease severity assessed by HY stage. In the MSA and PSP groups, no significant correlations were observed between the M/P ratio and age, which was consistent with a previous report [22]. In our results, there were no significant correlations between the test batteries, such as the M/P ratio on MRI and cardiac MIBG reuptake in all groups.

We previously reported the usefulness of the combined use of cardiac MIBG scintigraphy, olfaction and substantia nigra hyperechogenicity visualized by transcranial sonography to differentiate PD from MSA and PSP [7]. However, transcranial sonography is sometimes difficult to assess because of an insufficient bone window, especially in elderly Asian women [23]. In addition, olfactory function was also affected by cognitive impairment [[24], [25], [26]], so the clinical reliability of assessing olfactory function can be reduced when patients have dementia or significant cognitive impairment. On the other hand, the present tests using the M/P ratio and cardiac MIBG scintigraphy can be assessed even if patients have dementia or mild to moderate cognitive impairment. Thus, this combination should be of high utility in clinical practice as a supportive diagnostic marker of PD because up to 80% of PD patients develop dementia during the disease course [26].

Our study has several limitations. First, the sizes of the MSA and PSP groups were small, which could have impacted the overall findings. Second, two patients with PSP-PAGF, classified as atypical PSP, were included in our PSP cohort. As Sakurai et al. [27] reported that the degree of midbrain atrophy was not consistent in atypical PSP cases, unlike PSP-RS cases, future studies with larger cohorts are needed to validate the utility of the M/P ratio as it applies to patients with atypical PSP. Third, we lack of the healthy subjects, nevertheless M/P ratio might be influenced by age. However, it was difficult to conduct cardiac MIBG scintigraphy to healthy control. Fourth, although we found improvement in the sensitivity by using either the M/P ratio or cardiac MIBG scintigraphy revealed abnormalities, the usefulness of this battery may be limited in clinical practice because its low specificity. Therefore, when M/P ratio already showed abnormality, adding cardiac MIBG scintigraphy can be preferable because both positive results provide a satisfactory specificity in PD diagnosis (PD vs. MSA; 100%, PD vs. PSP; 90%).

In conclusion, we confirmed the M/P ratio alone was the potentially useful tool for differentiating PD from MSA and PSP. In addition, combined use of M/P ratio with cardiac MIBG scintigraphy could substantially improve its diagnostic power.
